# The Book World of Medicine and Science

**Published:** 1893-04-15

**Authors:** 


### The Book World of Medicine and Science.

### THE APRIL REVIEWS.

The Fortnightly Review contains an article by Professor Wallace on the subject lately debated by Mr. Herbert Spencer in the *Contemporary*, "Are Individually-Acquired Characters Inherited?" The writer deems all the alleged facts and arguments inconclusive, and that the balance of the evidence yet adduced is altogether in favour of such characters not being inherited. One or two new suggestions are offered to account for the old problem why a dog turns round three or four times before settling himself to sleep. Sir Richard Temple, M.P., gives an account of a proposed scheme for the superannuation of elementary teachers. He urges that teaching is an exceptionally wearing career, and one in which any loss of vigour is very speedily traced in results, and he considers that the teachers should largely contribute to, while the State should organise, control, enforce, and partially sustain, a system of superannuation pensions, commencing for women at sixty, and for men at sixty-five years of age. The pensions are to range from £45 to £90 a year, and the actuarial calculations, which are gone into at some length, are made on the non-returnable basis.

With reference to the recent stir that has been made with regard to the dangers to health under which the white lead manufacture is carried on, Mr. Barrett Lennard writes to complain of many exaggerated statements. He claims that but few deaths occur annually from lead poisoning, and that the dangers are not more than may be easily met by care on the part of the workmen. It has, however, become only too certain in the history of nearly every trade that precautions which are dependent on the workman's instinct of self-preservation are systematically disregarded. The whole subject of this manufacture cannot be too thoroughly investigated.

In the Century for this month a letter from Mr. Carey Lea gives a description of the results ensuing in Germany from a Commission to report on dangerous employments. By the application of scientific methods, and with wonderful skill and ingenuity, the dangers in many trades have been entirely abolished, and in others have been even turned to profitable account. By the use of silver instead of mercury in the mirror factory at Fürth the number of days of illness caused by mercurial poisoning has been reduced from 4,074 in 1885 to none in 1890. In the lead works at Tarnowitz all the furnaces have been connected with powerful ventilators, which draw out the fumes by exhaustion, and the number of sick days has been thus reduced to one-sixth. In the manufacture of mineral fertilisers where the phosphate rock contains fluor spar, vapours of hydrofluoric acid highly injurious to the lungs are disengaged when the rock is treated with sulphuric acid. It has been found, however, that by bringing the hydrofluoric vapours into combination this danger is abolished, and a new product—artificial cryolite—is obtained. Our manufacturers in the North might well take a lesson from Germany in the matter of fouling the streams by the drainage of factories. In many parts of Yorkshire still, in defiance of law, the water assumes its natural colour only on Sunday, and fish are unknown. In Germany the principal difficulty in purifying the water was in the beetroot sugar

**THE BOOK WORLD OF MEDICINE AND SCIENCE.—(*continued*).**

factories. Yet at Ströbnitz factory, which works up 70,000 tons of beets each season, and discharges its waste water at the rate of over 1,000 gallons a minute, this difficulty has been entirely overcome, and the waste, cleared of every injurious particle, flows off free from smell or taste. At a starch factory which formerly fouled the river Werra for a distance of some miles, the waste products have been found to be so valuable for fertilising purposes that it is impossible to meet the demand for them from the farmers.

Major-General Drayson has attracted a great deal of attention by his article in the *Nineteenth Century* on "The Art of Breathing." It seems after all that the fat boy in Pickwick was the true master of hygiene, and every one is to try to acquire his accomplishment of "breathing hard." General Drayson found that the faintness and palpitation induced by breathing the rarified air at a height of 7,000 feet above the sea, disappeared when he breathed more rapidly, and thus filled his lungs with a sufficiency of oxygen. He claims also that heartache, headache, and toothache may all be driven away by rapid breathing—from forty to fifty breaths a minute. Undoubtedly there is something in it, and much good may be effected if the guests at a dinner party simultaneously begin to breathe hard when the atmosphere becomes vitiated, and thus call the attention of the host to the need for ventilation. Prince Krapotkin, on "Recent Science," discusses meteorological progress, specially with reference to the theories of the American scientist Ferrel. He gives some account also of the production by Henri Moissan, the French Academician, of minute crystals of diamonds, and touches upon the question of the action of environment upon organisms. Mr. G. R. Humphrey gives an interesting account of the self-collected libraries of several working men with an analysis of books taken out in some of the free libraries, all tending to show that the working man appreciates good books when time and opportunity serve. It would be interesting to have as a pendant some analysis of the reading of *idle* men—the unemployed of Bond Street and Pall Mall—if such were attainable. How many works of "History and Science" in proportion to the latest novel?

